# A prospective, single-centered, cohort study comparing the treatment of renal stones by following PCNL types: Standard, tubeless & totally tubeless

**DOI:** 10.1016/j.amsu.2022.104325

**Published:** 2022-08-04

**Authors:** Mumtaz Ahmad, Hassan Mumtaz, Hassan-ul Hussain, Sharjeel Sarfraz, Manahil Rahat, Shamim Mumtaz

**Affiliations:** a Pakistan Association of Urological Surgeons, Pakistan; b Benazir Bhutto Hospital, Pakistan; c Maroof International Hospital, Pakistan; d Health Services Academy, Islamabad, Pakistan; e Dow University of Health Sciences, Karachi, Pakistan; f DHQ Hospital, Pakistan; g Oral Pathology: AFIP, Pakistan; h Islamabad Medical & Dental College, Pakistan; i Rawalpindi Medical University, Pakistan

**Keywords:** PCNL, Nephrolithotomy, Stone free rate, PERC, Endo urology

## Abstract

**Introduction:**

Renal stones are a frequent cause of morbidity globally. The number of lumbotomies performed for benign lithiasis has been greatly decreased with the usage of Percutaneous Nephrolithotomy (PCNL). Further development is aimed at reducing tract size, leading to numerous advanced minimally invasive PCNL procedures like mini-PERC, ultra-mini-PERC, and micro-PERC. The aim of this study was to evaluate whether tubeless or totally tubeless PCNL is the safest and most efficient, less morbid management technique for renal stones compared to the standard PCNL with a nephrostomy tube.

**Methodology:**

This is a comparative, prospective, single-centered, cohort study that took place between August 2015 and January 2018 in the Urology department of Benazir Bhutto Hospital in Rawalpindi, Pakistan. 218 patients having single/multiple stones of variable sizes were enrolled in the study. Participants were stratified into three groups; Group A: Standard PCNL treatment; Group B: Tubeless PCNL treatment; Group C Totally Tubeless treatment. Mean operation time (±SD) and stone-free rates were our primary outcomes. The rate of complications during and post-operative complications were our secondary outcomes.

**Results:**

A total of 181 patients were included in our study. A decreasing trend can be seen in mean operation time as we move from Group A to Group C *(p* = *0.000)*. The rate of problems during operation in each group was highest (45.8%) in Group A, much lesser problems in Group C (13.3%), and least problems in Group B (8.1%) *(p* = *0.000)*. The postoperative complication rate was again the highest in Group A (30.5%), low in Group C (8.3%), and extremely low in Group B (1.6%)

**Conclusion:**

Tubeless PCNL proved to be the safest and most effective when compared to standard and totally tubeless PCNL procedures. It also showed the highest stone-free rates and least ‘unsatisfactory’ results amongst all the groups. Conclusively, it should be performed in routine preferably.

## Highlights

• In terms of safety and efficacy, tubeless PCNL outperformed both regular and completely tubeless PCNL.
• The Tubeless PCNL is much superior than other techniques of PCNL.
• Aftercare and difficulties during surgery are virtually nonexistent with tubeless PCNL.
• Stone-free rates were also the highest and the least “unsatisfactory” across all groups. Finally, it's best if it's done on a regular basis.

## 1 Introduction

Humans are commonly susceptible to urinary tract stones, especially renal stones whose prevalence has substantially inclined in the last few decades. Renal stones are a frequent cause of morbidity globally. The rate of lifetime recurrence of renal stones is found to be 50% [1,2]. Pakistan is amongst those countries where the prevalence of renal stones is comparatively higher [3] specifically, in the areas of Northern Sindh and Southern Punjab [4]. The majority of the population resides in rural areas where the climatic conditions are moderate to extremely hot especially from April to September. Additionally, the population does not follow the recommended fluid intake instructions. Consequently, both of these factors lead to increased chances of renal stone formations [5].

In 1995, Joseph Y. Clark estimated that urolithiasis in the United States costs $1.83 billion annually [6]. Stones' economic burden is shifting from inpatient to outpatient care, according to data. Medical office visits and emergency room visits increased from 43% in 1994 to 53% in 2000 in expenditures on stone disease [7]. Saigal and colleagues found that kidney stones had a large economic impact in 2005, with annual estimates reaching $5 billion [8]. A study by Omar a Raheem and colleagues in 2017 found that Urinary stone incidence and prevalence are increasing over the world. Climate change, nutritional shifts, and obesity are among the most common explanations. PCNL has grown increasingly popular because of lower recurrence rates and costs. Advances in technology have made it easier and less expensive to treat kidney stones [9].

The number of lumbotomies performed for benign lithiasis has been greatly decreased with the usage of Percutaneous Nephrolithotomy (PCNL) [10,11]. In comparison with the Extracorporeal Shock Wave Lithotripsy (ESWL) method, PCNL is way more effective for treating <2 cm renal stones [10]. However, there have been reported incidents of post-operative pain, urine leakage, infections, prolonged hospitalization, and other complications with the use of nephrostomy catheters [12,13]. In order to overcome this, numerous modifications have been made to PCNL techniques to reduce post-operative pain, faster recovery and less hospitalization stay. Such advancements include the use of ureteric stents (JJ stents) instead of nephrostomy catheters which came to be known as tubeless PCNL [14,15].

However, usage of JJ stent in tubeless PCNL was also associated with stent-related issues like nocturia, pain, urgency, frequency, and hematuria [16]. Consequently, some studies preferred not using even the JJ stents, but only ureteric catheters, and this was called totally tubeless PCNL [12,13]. Reduced rates of morbidity are associated with reduced tract size [17], consequently, further development is aimed at reducing tract size, which led to numerous advanced minimally invasive PCNL (MIP) procedures like mini-PERC [18], ultra-mini-PERC [19], and micro-PERC [20].

**Rationale:** Despite being a global threat, very few randomized controlled trials with smaller sample sizes have been conducted in Pakistan comparing either of the two or three of the standard, tubeless, and totally tubeless techniques. There seemed a dire need to conduct a trial comparing all three techniques together to conclude efficient, time-saving, and safer renal stone removal treatments.

**Objectives:** The aim of this study was to evaluate whether tubeless or totally tubeless PCNL is the safest and most efficient, less morbid management technique for renal stones compared to the standard PCNL with a nephrostomy tube.

## 2 Methodology

This is a comparative, prospective, single-centered cohort study that took place between August 2015 and January 2018 in the Urology department of Benazir Bhutto Hospital in Rawalpindi, Pakistan. 218 patients having single/multiple stones of variable sizes were enrolled in the study. A sample size of 197 patients was calculated by Rao soft software. We kept the confidence interval at 95% with a 5% margin of error, and a 50% response distribution. Approval from the ethical committee was taken for the process of case selection. Participants were stratified into three groups; Group A: Standard PCNL treatment; Group B: Tubeless PCNL treatment; Group C Totally Tubeless treatment. Our study is fully compliant with the STROCSS 2021 guidelines [21]. A complete STROCSS 2021 checklist has been provided as a supplementary file. Our study has been registered on Research Registry with the following UIN: researchregistry7965 [22]. Our study is in accordance with the Declaration of Helsinki.

**Inclusion criteria:** Patients above the age of 4 having single/multiple renal stones with a stone's maximum diameter of <5.5 cm in coronal images on CT scans were included in the study.

**Exclusion criteria:** Patients with uncorrectable bleeding disorders, febrile urinary tract infections, single-functioning kidney, musculoskeletal deformities, acute infections, and cardiovascular and pulmonary comorbidities were excluded from the study.

Patients undergoing PCNL have post operative bleeding complication, therefore patients with uncorrectable bleeding disorders eg Von Willebrand disease, Hemophilia & Vitamin K Deficiency were excluded to assess the post operative complications and risk.

14 patients from Group A, 13 patients from Group B, and 10 patients from Group C were excluded.

### 2.1 PCNL techniques

Complete blood count, serum urea and creatine levels, and urine analysis were performed before the procedure. Ultrasonography was performed to evaluate the radiopacity of stones. A 5-Fr ureteric catheter was inserted into the ureter through cystoscopy with the patient lying in a lithotomy position. The patient was then asked to lay in a prone position. The chosen site was then punctured using an 18-G access needle guided by fluoroscopy after which a guidewire was inserted. Dilations of the tract were performed through Amplatz dilators and a 30F Amplatz sheath was placed after which stone fragmentation was performed by different modalities.

In all the groups, either pneumatic or ultrasonic lithotripsy or both were performed. All the procedures were performed under general anesthesia.

In Group A, after the procedure, a 22-Fr nephrostomy tube was inserted into the pelvicalyceal system along with either a ureteral stent (JJ stent) or a ureteric catheter. In Group B, a JJ stent was inserted after the procedure without nephrostomy with an access sheath size of 12-Fr. In Group C, only a ureteric catheter was inserted after the procedure with an access sheath size of 4.8-Fr. A nephrostomy tube was not used in Groups B and C.

Mean operation time was considered from the puncture of the site till the end of the procedure. In order to evaluate the stone-free rate, CT scans were done after two weeks of follow-up. Patients with no symptoms having ≤2 mm residual stone fragments or a negative CT scan were considered to have a stone-free status/full clearance. Stone scoring was done through Guy's stone scores to predict the success rates following PCNL treatments. Discharged patients were guided to return to the emergency department in case of any post-operative complications. Clavien-Dindo Classification was used to rate the postoperative complications [19]. Written informed consent has been obtained from each participant before the study.

### 2.2 Primary outcomes

Mean operation time (±SD) and stone-free rates were our primary outcomes.

### 2.3 Secondary outcomes

The rate of complications during and post-operative complications were our secondary outcomes.

**Statistical analysis:** We analyzed the collected data using SPSS version 24 (IBM, Armonk, New York, USA). Descriptive statistics (frequencies) were used to describe and compare the demographic details amongst the groups. Secondly, differences between the means of continuous variables were evaluated by conducting Independent Sample T-tests. Thirdly, Pearson's chi-squared tests were performed to determine the association between the type of PCNL and categorical variables. We considered the P-value as significant if it was <0.05.

## 3 Results

A total of 181 patients were included in our study. Group A consisted of 59 participants while Group B and C had 62 and 60 participants respectively. The mean ages of the population were 37.6, 34.7, and 32.3 in Group A, B, and C respectively. There were more males than females in Groups A and C while females predominated in Group B. The mean stone size was 2.1 cm in Group A, 1.9 cm in Group B and 1.6 cm in Group C. Mean hemoglobin, urea, and creatinine levels did not fluctuate much among the three groups. Total leukocyte count was found to be a little lower in Group A when compared to other groups (Table 1).

**Table 1:** Demographic details of patients and stone-related traits.

	Group A	Group B	Group C
**Total patients (n** = **181)**	59	62	60
**Age – yrs**	37.6 ± 13.2	34.7 ± 14.9	32.3 ± 11.9
**Sex (M/F)**	37/22	30/32	36/24
**Stone size (cm)**	2.1 ± 0.8	1.9 ± 1.0	1.6 ± 0.3
**Hemoglobin (mg/dl)**	14.1 ± 1.9	13.3 ± 1.6	13.9 ± 2.6
**TLC (×10**^9^**/L)**	7.5 ± 1.9	7.8 ± 1.7	8.1 ± 2.2
**Urea (mg/dL)**	30.5 ± 9.7	29.5 ± 11.1	27.7 ± 13.3
**Creatinine (mg/dL)**	0.9 ± 0.3	0.9 ± 0.4	0.9 ± 0.6
**Chief complaints (Right/Left/Bilateral pain)**	24/24/5	30/24/4	27/30/1
**Radiopacity (%)**	98.3	98.4	100
**Location (Pelvis/Upper Pole/Middle Pole/Lower Pole/PUJ/All calyces)**	25/0/0/1/0/4	24/1/1/7/6/0	31/0/0/6/2/5
**Urine analyses (Normal/Pyuria/Hematuria)**	31/11/11	43/7/4	40/6/3

The majority of the patients had chief complaints of right lumbar region pain and left lumbar region pain in all three groups, while a few had complaints of bilateral lumbar region pain, lower urinary tract symptoms, and acute urinary retention. Almost all of the stones showed radiopacity on x-ray in all the groups. The most common location of the stone in all the groups was the renal pelvis, followed by the lower calyx. Other less common locations included upper calyx, middle calyx, all calyces, pelvic ureteric junction, proximal ureter, distal ureter, and bladder. For the majority of the participants, urine analyses were found to be normal, while the frequency of pyuria was almost twice the frequency of hematuria in Groups B, and C. However, Group A had the same number of patients (n = 11) having hematuria and pyuria. A simultaneous finding of pyuria and hematuria was reported in a minority of the participants of all groups (Table 1).

Table 2 shows various comparisons among the groups. A decreasing trend can be seen in mean operation time as we move from Group A to Group C with the highest mean operation time in Group A (66.4 min) and lowest in Group C (34.1 min) which significantly associates the type of PCNL with operation time *(p* = *0.000)*. The procedure side chosen for operation was the left side in more than half of the participants in Group C. In Group A, the right side was more commonly used while Group B comprised an equal number of patients for the right and left sides. Retrograde catheterization was successfully performed in all the patients of Group C. However, it was successfully done in around 88% of the patients in Groups A and B, failing in the minority of them. The middle pole of the kidney was the most punctured site in Groups C and D while the lower pole was targeted more in Group A. The least common was the upper pole in all the groups.

**Table 2:** Comparison of operating time, problems, procedure side and site, modality, and Guy's Stone Score.

	Group A	Group B	Group C	P-value
**Operation time (mins)**	66.4 ± 29.3	36.3 ± 16.8	34.1 ± 19.5	0.000
**Procedure side (Right/Left)**	31/28	31/31	24/36	–
**Retrograde catheterization(Yes/No/Failed)**	52/1/4	54/2/4	60/0/0	–
**Puncture site(Upper pole/Mid pole/Lower pole)**	9/19/30	12/28/22	9/28/20	–
**Rate of problems during operation (%)**	45.8	8.1	13.3	0.000
**Stone fragmentation Modality(Pneumatic/Ultrasonic)**	25/24	0/0	0/0	–
**Guy's Stone Score (1/2/3/4)**	30/17/7/5	35/18/5/4	32/15/7/6	–

Table 2 also compares the rate of problems during operation in each group with the highest (45.8%) in Group A, much lesser problems in Group C (13.3%), and least problems in Group B (8.1%) which significantly associates problems during operation with the type of PCNL *(p* = *0.000)*. Only pneumatic and ultrasonic lithotripsy were used for stone fragmentation in all the groups. The highest number of participants (n = 35, n = 18) with GSS-1 and GSS-2 were found in Group B, while the number of patients with GSS-3 and GSS-4 was found to be almost equal in all groups.

Table 3 displays comparisons of postoperative outcomes, postoperative complications, stone-free rates, and Clavien score percentages. Results rated as ‘good’ increased significantly as we move from Group A to C while ‘unsatisfactory’ results were least in Group B *(p* = *0.001)*. Stone-free rate showing clearance of fragments after the procedure was highest in Group B (74.2%) and lowest in Group A (44.1%), again indicating a significant association between SFR and type of PCNL *(p* = *0.001)*. The postoperative complication rate was again the highest in Group A (30.5%), low in Group C (8.3%), and extremely low in Group B (1.6%) manifesting an extremely significant association *(p* = *0.000)*. The distribution of postoperative complications according to Clavien-Dindo classification in Table 3 significantly shows that scale of scores was comparatively higher in Group A *(p* = *0.000)*.

**Table 3:** Postoperative outcomes, complications, SFR and Clavien Score percentages.

	Group A	Group B	Group C	P-value
**Result (Good/Satisfactory/Unsatisfactory)**	29/27/3	46/15/1	50/8/2	0.001
**SFR (%)**	44.1	74.2	68.3	0.001
**Complication rate (%)**	30.5	1.6	8.3	0.000
**Clavien Score 1**–**2 n (%)**	20 (33.9)	4 (6.5)	7 (11.7)	0.000
**Clavien Score 3a-5 n (%)**	4 (6.8)	3 (4.8)	0 (0)	

Table 4 indicates the relation of GSS with SFRs and results. An extremely significant inverse relationship can be seen between increasing GSS with decreasing SFRs *(p* = *0.000)*. Good and satisfactory results decreased while unsatisfactory results increased significantly with increasing GSS *(0.015)*.

**Table 4:** Relating GSS with results and SFR percentages.

Guy's Stone Score	GSS-1	GSS-2	GSS-3	GSS-4	P-value
**SFR (%)**	87.4	52.4	48.4	4.3	0.000
**Results (Good/Satisfactory/Unsatisfactory)**	70/32/1	59/24/1	14/14/3	17/4/2	0.015

Table 5 compares the problems faced during the operation and post-operative complications amongst the groups. Access to the stones and/or stone removal, intraoperative bleeding, and problem with ureteric catheterization were the most common issues. Post-operative abdominal pain, fever, and nephrostomy side leakage were the most prevalent postoperative outcomes.

**Table 5:** Problems during operation and postoperative complications.

		Group A	Group B	Group C
**Problems during operation n (%)**	**Stone access and/or removal**	16 (27.1)	4 (6.5)	6 (10)
	**Intra-operative bleeding**	4 (6.8)	0 (0)	1 (1.7)
	**Ureteric catheterization**	6 (10.2)	0 (0)	0 (0)
	**Retroperitoneal leakage**	1 (1.7)	1 (1.6)	1 (1.7)
**Postoperative complications n (%)**	**Bleeding**	1 (1.7)	0 (0)	0 (0)
	**Fever**	3 (5.1)	1 (1.6)	3 (5)
	**Abdominal pain**	3 (5.1)	0 (0)	1 (1.7)
	**Nephrostomy side leakage**	5 (8.5)	0 (0)	0 (0)
	**Blood transfusion**	1 (1.7)	0 (0)	0 (0)
	**Pyelonephritis**	1 (1.7)	0 (0)	0 (0)
	**Fever + leakage**	3 (5.1)	0 (0)	0 (0)
	**Abdominal pain + leakage**	1 (1.7)	0 (0)	0 (0)
	**Dyspnoea**	0 (0)	0 (0)	1 (1.7)

## 4 Discussion

Our study results show that Tubeless PCNL is safest and most effective when compared to standard and totally tubeless procedures. It also showed the highest stone-free rates and least ‘unsatisfactory’ results amongst all the groups. Not a significant difference was seen in mean operating time between tubeless and totally tubeless techniques.

The increasing frequency and recurrence of urolithiasis in people is making it a worldwide threat [23]. There is a dire need to stick to the most efficient, time-saving, cost-effective technique with reduced postoperative complications. With increasing modifications in PCNL techniques, a decline in morbidity rate has been reported [24]. The benefits of postoperative nephrostomy tube are to prevent renal drainage, prevent bleeding, and provide access for redo-PCNL while decreasing morbidity [25]. However, in recent years, attempts have been made to alter the conventional PCNL in order to reduce morbidity and postoperative complications [26]. To lessen the discomfort and tube-related morbidity, different modifications have been made, like the use of a JJ stent [14,15] or not using the tube at all [12,13]. The advanced approach should be to make the PCNL more convenient and cost-effective for the patients without risking their safety and efficacy of treatment.

In our study, statistically significant differences are reported amongst the three groups regarding mean operation time, rate of complications during and after the procedures, stone clearance rate, the satisfaction of results, GSS, and Clavien-Dindo scores. Our study indicates lesser mean operation time taken in minimally invasive techniques especially tubeless as compared to standard PCNL which is also shown in studies by Qadir et al. [25]. Aghamir et al. [12] and Istanbulluoglu et al. [16] showed no significant difference in mean operating time between standard and totally tubeless techniques. While contrary results are shown in studies by Guddeti et al. [27] and Bozzini et al. [28] where standard PCNL took lesser time to operate. In our study, the rate of problems during operation and postoperative complications was significantly higher in standard PCNL when compared to other techniques. Totally tubeless was second on the list while the least complications were faced in tubeless PCNL. Bozzini et al. [28]and Istanbulluoglu et al. [16] also showed that standard PCNL had the highest complication rates. Totally Tubeless PCNL showed higher complication rates than tubeless PCNL in our study, which is contradictory to the findings in a study presented in the European Association of Urology, in which lesser pain, infections, hematuria, fever were associated with totally tubeless PCNL as compared to tubeless PCNL [29].

Comparatively better results (least unsatisfactory results) are seen in tubeless PCNL in our study as compared to standard and totally tubeless PCNL. According to Ahmed et al., totally tubeless PCNL is the most effective technique [29], while another study [27] suggested that tubeless PCNL is the safest of all in patients with no major intra-operative hemorrhage but totally tubeless is the best for patients having no residual stones. Desai et al. concluded that tubeless PCNL was better when they compared it with conventional large and small-bore PCNL [30]. However, Paul and associates [31] concluded that to perform a tubeless technique, a strict inclusion criterion needs to be decided. According to other studies [15,[30], [31], [32], [33], [34]], tubeless PCNL is preferred due to safety and efficiency even if the patients have deranged kidney functions or have a single kidney or those in need of bilateral simultaneous PCNL or with two to three access tracts or supracostal access.

SFRs were found to be much higher in totally tubeless and tubeless PCNL as compared to the standard technique. A meta-analysis by Li et al. showed no significant difference in SFRs between standard and totally tubeless PCNL [35]. Another meta-analysis showed no significant difference in SFRs between standard and tubeless PCNL [36]. On the other hand, contrary results are reported by a study [29] where higher SFR was seen in standard PCNL.

To predict the success rate and complications following PCNL, GSS is used. According to a study done in Thailand, the success rates were found to be 87.5% for GSS-1, 71.4% for GSS-2, 53.6% for GSS-3, and 38.5% for GSS-4 [37]. These results are similar to those of our study, as GSS-1 had the highest success rate of 87.4%, GSS-2 had 52.4%, GSS-3 had 48.4% and GSS-4 showed the lowest success rate of 4.3%. Clavien-Dindo classification in our study was also comparable to the Thailand study [37], as major complications (Clavien score of 1–2) were significantly higher in the cases with a higher GSS while minor complications (Clavien score of 3a-5) were significantly lower in patients with lower GSS. Tubeless PCNL entails a benefit over the standard PCNL in terms of shorter hospital stay and early recovery time in patients [38]. A shorter hospital stay renders tubeless PCNL a better procedure as it can cut short the healthcare expenditure. Early mobilization after the tubeless PCNL technique can dispense better health outcomes in the patients.

### 4.1 Strength

Our study being one of a kind according to our knowledge compared all three techniques of PCNL procedures which were lacking in the already present literature.

### 4.2 Limitations

The limitations of our study are the small sample size and long-term complications of PCNL procedures.

## 5 Conclusion

Tubeless PCNL proved to be the safest and most effective when compared to standard and totally tubeless PCNL procedures. Not a significant difference was seen in mean operating time between tubeless and totally tubeless techniques, but tubeless PCNL proved to be much better than other techniques in other areas. Tubeless PCNL does not carry any significant risk of postoperative complications and problems during operation. It also showed the highest stone-free rates and least ‘unsatisfactory’ results amongst all the groups. Conclusively, it should be performed in routine preferably.

## Funding

The authors declare no funding

## Ethical approval

Ethical approval was obtained from the ethical committee of Benazir Bhutto Hospital Rawalpindi Ref ERC: BBH-UROL/CERT-01092021/24820.

## Consent

The informed consent from the patients was obtained considering Helsinki's Declaration.

## Author contribution

The main concept was determined by Mumtaz Ahmad, Collection of data is done by Sharjeel Sarfraz, Data is interpreted by Manahil Rahat, Writing of the manuscript is done by Hassan Mumtaz, Hassan-ul-Hussain, Manuscript editing is done by Shamim Mumtaz.

## Guarantor

Mumtaz Ahmad & Hassan Mumtaz.

## Provenance and peer review

Not commissioned, externally peer-reviewed.

## Declaration of competing interest

The authors declare no conflicts of interest.
